# Supplementary material for: Aligning policymaking in decentralized health systems: Evaluation of strategies to prevent and control non-communicable diseases in Nigeria
Source: PLOS Glob Public Health. 2021 Nov 10;1(11):e0000050. doi: 10.1371/journal.pgph.0000050 (PMC10022121; doi:10.1371/journal.pgph.0000050)
Supplement: S1 File — (DOCX) [file pgph.0000050.s001.docx]

**In-depth interview guide with policy makers (FMOH, WHO and NPHCDA NCDs program coordinators)**

**The aim of the Interview**

This interview is part of the research study “a health system approach to understanding and improving the delivery of care for non-communicable diseases (NCDs) at the primary health care level through the team of healthcare workers”. This study is being conducted as part of fulfilment of PhD programme with The George Institute for Global Health Australia and the UNSW, Sydney to explore the roles of the Primary Health Care workers for the prevention and management of non-communicable diseases like cardiovascular diseases and Diabetes mellitus and their risk factors.

**FMOH (NCDs and Cancer Control Unit) – Interview Guide**

| **Q. No** | **Main question/exploratory questions** | **Probing questions** | **Tell me more about** |
| --- | --- | --- | --- |
| 1 | **Policy Context**   - What is your opinion about the development of the National Policy and Strategic Plan on NCDs in Nigeria (2013 & 2015) - What were the issues within the Nigeria context that led to development of the policy - To what extent were the states, local governments, civil society organisations and the community involved in the formulation of this policy/strategy? | What was the rationale for formulating the policy/strategy?  (Probe for whether there were political changes, health sector reforms, organizational changes, fiscal policies, and changes in government  What was your role in the formulation of the policy? | For National Cancer plan, ask about the integration of primary prevention into PHC |
| 2 | **Policy Implementation (at the State and PHC level)**   - To what extent have the policies/strategy been implemented (a) at National level (b) state and local government (PHC) levels - What were the roles played by the FMOH to ensure state level implementation - Can you please describe your experience as you participated in the implementation of the policy at the state level - What inputs and resources were required to implement the policy - What factors enabled the implementing these policies at the state level? - What were the challenges encountered in implementing the policies - Is there any accountability framework in place to ensure policy implementation at the state level? - What would you recommend facilitating the implementation of policies for the state level for the future? | - What proportion of states have adopted and implemented the policy and strategies - Do you think the policy implemented according to the policy requirements - What key activities were completed during the policy implementation? - Did these activities result in the anticipated outputs? - What in your opinion went well? - What could have been done differently? - Were these inputs and resources made available? - What other (external) factors influenced the implementation? - Were there any unintended consequences? - Who monitors it? How effective and useful has it been? - Probe for recommendations to facilitate the process of policy implementation | - Who led the process of the policy implementation at the state level - Which other sectors/stakeholders were involved in the implementation? - Was the policy implemented across the states consistently? - (Probe: For amount of funding; sources of funding; technical support, advocacy/stakeholders’ involvement, supervision, monitoring, etc) |

**In-depth interview guide with policy makers (State officials’ questionnaire)**

**The aim of the Interview**

This interview is part of the research study “a health system approach to understanding and improving the delivery of care for non-communicable diseases (NCDs) at the primary health care level through the team of healthcare workers”. This study is being conducted as part of fulfilment of PhD programme with The George Institute for Global Health Australia and the UNSW, Sydney to explore the roles of the Primary Health Care workers for the prevention and management of non-communicable diseases like cardiovascular diseases and Diabetes mellitus and their risk factors.

**Interview guide for State officials’ questionnaire**

| **S.No** | **Main question/exploratory questions** | **Probing questions** | **Tell me more about** |
| --- | --- | --- | --- |
| 1 | **Implementation process *(****The structures, resources and mechanisms through which delivery is achieved)*   - Are you aware of the (i) National Policy and Strategic Plan on NCDs in Nigeria (2013 & 2015) and (ii) Nigeria National Cancer Control Plan? - Has your state implemented the strategy/policy? - How was the implementation achieved/carried out - What inputs and resources were required to implement the policy? | - What is your opinion about the policy/strategy development in this country? - What do you think was the rationale for formulating the policy/strategy - What were the roles played by your organization to ensure implementation - What key activities were completed during the policy implementation? - Probe for amount of funding; sources of funding; technical support, advocacy/stakeholders’ involvement, supervision, monitoring, etc | - Was your state involved in the formulation of the policy/strategy? - Probe for advocacy, stakeholders’ involvement, the roles of FMOH/NPHCDA and other partners - Were these inputs and resources made available? |
| 2 | **Consistency of what is implemented with the planned intervention)**   - Did your state implement the policy/strategy as required in document | - What factors made it possible (or impossible) for the implementation of the policy/strategy as it is. | - If no, go to question 3 |
| 3 | **Alterations made to implementation to achieve better contextual fit)**  How did your state adapt this policy/strategy to fit into the context of your state   - What factors influenced the adaptation into this context - What key activities were completed during the policy adaptation - Which of the following strategic areas were adapted?  1. Advocacy 2. Leadership and governance 3. Multi-sectoral action and partnership 4. Promotion of healthy lifestyle 5. Strengthening and reorientation of the health system 6. Promotion and support of national capacity for quality research 7. Monitoring and evaluation of trends and determinants of NCDs | - How were the decisions to the adaptation made? Was the FMOH involved in the adaptation? Was FMOH updated with the adapted version? - probe for political, financial, human resources, priority of other programmes – MNCH, communicable diseases, etc |  |
| 4 | **How much intervention is delivered;**   - To what extent has the adapted policy/strategy been implemented? - What factors enabled the implementing these policies in your state? - What were the challenges/barriers encountered in implementing the policies. - What would you recommend facilitating the implementation of policies for the state level for the future? | - Were there any unintended consequences? - Probe for recommendations to facilitate the process of policy implementation | - What other (external) factors influenced the implementation? |
| 5 | **Extent of contact of implementation with target audience**  Did these activities result in the anticipated outputs?   1. Do you have a designated focal point responsible for the coordination of NCDs prevention and control?    1. At State level    2. At the LGA level 2. Do you have a budget line and adequate resources to support NCDs prevention and control    1. At State level    2. At the LGA level 3. Have you conducted (or been conducting) capacity building for the implementation of the policy/strategy    1. At State level    2. At the LGA level 4. Have you conducted advocacy and social mobilization for the prevention and control of NCDs    1. At State level    2. At the LGA level 5. Is there a mechanism in place to ensure access to essential medicines, basic technologies, consumables and services for the prevention and control of NCDs    1. At State level    2. At the LGA level 6. Is there a mechanism in place to ensure effective linkages and referrals between Primary Health Care and higher levels of care 7. Is there a mechanism in place to promote appropriate partnerships in consultation with the Federal Ministry of Health to prevent and control NCD 8. Has data management on NCDs been included in the integrated disease surveillance and response (IDSR) 9. Is there a mechanism in place for effective implementation, supervision, monitoring and evaluation of this plan of action    1. At State level    2. At the LGA level | |  |
