# Supplementary material for: Aligning policymaking in decentralized health systems: Evaluation of strategies to prevent and control non-communicable diseases in Nigeria
Source: PLOS Glob Public Health. 2021 Nov 10;1(11):e0000050. doi: 10.1371/journal.pgph.0000050 (PMC10022121; doi:10.1371/journal.pgph.0000050)
Supplement: Attachment — Submitted filename: Response to Reviewers Comments_31-8-2021.docx [file pgph.0000050.s002.docx]

The George Institute for Global Health,

Level 5, 1 King St, Newtown, NSW 2042, Australia

31^st^ August, 2021

**Dear Oliver Ian Tolentino,**
PLOS Global Public Health

**Re: Edits requested on your submission PGPH-D-21-00385R1 [EMID:c421eec19bde6b9b]: *“Aligning policymaking in decentralized health systems: evaluation of strategies to prevent and control non-communicable diseases in Nigeria” –* Response to reviewers’ comments**

Thank you for checking our manuscript and giving us the opportunity to edit it further. We have responded to the comments you and revised the manuscript accordingly.

**RESPONSE TO COMMENTS**

1. Please amend your detailed Financial Disclosure statement. This is published with the article, therefore should be completed in full sentences and contain the exact wording you wish to be published.
   i. State what role the funders took in the study. If the funders had no role in your study, please state: “The funders had no role in study design, data collection and analysis, decision to publish, or preparation of the manuscript.”

**RESPONSE:**
We have updated this by adding *“The funders had no role in study design, data collection and analysis, decision to publish, or preparation of the manuscript.”*

2. Please ensure that the funders and grant numbers match between the Financial Disclosure field and the Funding Information tab in your submission form. Note that the funders must be provided in the same order in both places as well. Currently, the only information in the Funding Information: Funder Name: the george institute for global health, Grant Number: Seed Grant funds dedicated for under-served populations in LMICs for 2019/2020, Grant Recipient: Associate Professor Rohina Joshi

**RESPONSE:**
This has been amended.

Also submitted along with this response is a clean and tracked change manuscript.

Please do not hesitate to contact me should you require any further clarification.

Yours sincerely,

**Whenayon Simeon Ajisegiri**

Tel.: +61469238057

E-mail: wajisegiri@georgeinstitute.org.au
